# The impact of concomitant pulmonary infection on immune dysregulation in *Pneumocystis jirovecii* pneumonia

**DOI:** 10.1186/1471-2466-14-182

**Published:** 2014-11-19

**Authors:** Chung-Wei Chou, Fang-Chi Lin, Han-Chen Tsai, Shi-Chuan Chang

**Affiliations:** Institute of Clinical Medicine, National Yang-Ming University, Taipei, Taiwan; Department of Medical Affairs, Taipei Municipal Gan-Dau Hospital, Taipei, Taiwan; Department of Chest Medicine, Taipei Veterans General Hospital, No. 201, Section 2, Shih-Pai Road, Taipei 112, Taiwan; School of Medicine, National Yang-Ming University, Taipei, Taiwan; Department of Nursing, Taipei Veterans General Hospital, Taipei, Taiwan; Institute of Emergency and Critical Care Medicine, National Yang-Ming University, Taipei, Taiwan

**Keywords:** Anti-inflammatory cytokines, Bronchoalveolar lavage fluid, Non-acquired immunodeficiency syndrome, *Pneumocystis jirovecii* pneumonia, Pro-inflammatory cytokines

## Abstract

**Background:**

Concurrent infection may be found in *Pneumocystis jirovecii* pneumonia (PJP) of non-acquired immunodeficiency syndrome (AIDS) patients, however, its impact on immune dysregulation of PJP in non-AIDS patients remains unknown.

**Methods:**

We measured pro-inflammatory cytokines including tumor necrosis factor (TNF)-α, interleukin (IL)-1β, IL-8, IL-17, monocyte chemoattractant protein-1 (MCP-1) and anti-inflammatory cytokines including IL-10 and transforming growth factor (TGF)-β1 and IL-1 receptor antagonist (IL-1RA) and inflammatory markers including high mobility group box 1, Krebs von den Lungen-6, receptor for advanced glycation end product, advanced glycation end product, surfactant protein D in bronchoalveolar lavage fluid (BALF) and blood in 47 pure PcP and 18 mixed PJP and other pulmonary infections (mixed PJP) in non-AIDS immunocompromised patients and explored their clinical relevance. The burden of *Pneumocystis jirovecii* in the lung was determined by counting number of clusters of *Pneumocystis jirovecii* per slide and the concentration of β-D-glucan in BALF. PJP severity was determined by arterial oxygen tension/fraction of inspired oxygen concentration ratio, the need of mechanical ventilation and death.

**Results:**

Compared with pure PJP group, mixed PJP group had significantly higher BALF levels of IL-1β, TNF-α and IL-8 and significantly higher blood levels of IL-8. The BALF ratios of TNF-α/IL-10, IL-8/IL-10, IL-1β/IL-10, TNF-α/TGF-β1, IL-8/TGF-β1, IL-1β/TGF-β1 and IL-1β/IL-1RA were significantly higher in mixed than in pure PJP patients. There was no significant difference in clinical features and outcome between pure and mixed PJP groups, including inflammatory biomarkers and the fungal burden. In pure PJP patients, significantly higher BALF levels of IL-8 and the ratios of IL-8/IL-10, IL-1β/TGF-β1, MCP-1/TGF-β1, MCP-1/IL1RA and IL-8/TGF-β1 were found in the patients requiring mechanical ventilation and in non-survivors.

**Conclusions:**

In summary, concurrent pulmonary infection might enhance immune dysregulation of PJP in non-AIDS immunocompromised patients, but did not affect the outcome as evidenced by morbidity and mortality. Because of limited number of cases studied, further studies with larger populations are needed to verify these issues.

**Electronic supplementary material:**

The online version of this article (doi:10.1186/1471-2466-14-182) contains supplementary material, which is available to authorized users.

## Background

*Pneumocystis* is an opportunistic fungal pathogen that causes *Pneumocystis jirovecii* pneumonia (PJP). PJP-related morbidity and mortality appear to be a major health problem for patients with acquired immunodeficiency syndrome (AIDS) and for those with immunosuppression resulted from chemotherapy, organ transplantation and long-term treatment with steroid or other immunosuppressants for a variety of diseases [1]. The clinical features, radiological findings, response to treatment and outcome of PJP are reported to be widely different between the patients with or without AIDS [2, 3]. The reasons underlying the differences in clinical features, radiological findings, treatment response and outcome of PJP between the patients with and without AIDS remain to be elucidated.

Some studies suggest that pathology of PJP inflammatory response is the main factor attributed to morbidity and mortality of PJP patients [4–8], although pathology itself and underlying disorders resulting in impaired immune function are also of clinical importance. Our and previous studies [9, 10] indicated that immune dysregulation was found in PJP of the patients with AIDS and non-AIDS, and certain pro-inflammatory cytokine/anti-inflammatory cytokine ratios in bronchoalveolar lavage fluid (BALF) were of considerable value in assessing the severity of PJP and outcome of the patients [10].

Mixed pulmonary infections including PJP and other pathogens are uncommon in patients with AIDS or non-AIDS immunocompromised hosts. In this study, we intended to explore the impact of concomitant pulmonary infection on immune dysregulation of PJP as evidenced by the changes in pro-inflammatory cytokines and anti-inflammatory cytokines and its clinical relevance. Our study results showed concurrent pulmonary infection might enhance immune dysregulation of PJP in non-AIDS immunocompromised patients, but did not affect the outcome as evidenced by morbidity and mortality.

## Methods

### Non-AIDS PJP patients

Sixty five consecutive non-AIDS immunocompromised patients with PJP diagnosed by identification of *Pneumocystis jirovecii* cysts or trophozoites in Papanicolaou- and Gomori methenamine silver stained-smears of BALF at Taipei Veterans General Hospital from November 1987 to September 2012 were included for this study. The patients were classified into two groups. One group included patients with pure PJP. The other group included those with PJP and concomitant pulmonary infection including cytomegalovirus (CMV) pneumonia and/or co-infections with other pathogens. Peripheral blood samples were collected after bronchoalveolar lavage (BAL) for measurements of cytokines and inflammatory biomarkers. The institutional Review Board of Taipei Veterans General Hospital approved the study (No: 201009019IC and No: 96-04-02A), and written informed consent was obtained from all patients before entering the study of cytokines and inflammatory biomarkers in BALF and blood.

### Diagnostic criteria

Bacterial pneumonia was diagnosed when a quantitative bacterial culture from BALF samples grew pathogenic bacteria >10^4^ colonies/mL. CMV was considered to be the pathogen if it was demonstrated on BALF cytology smears or positive result of CMV culture from BALF. Fungal pneumonia was diagnosed when one of the following criteria was met: (1) fungi were identified by microbiologic studies in at least two different lower respiratory tract secretions including BALF; (2) fungi were demonstrated by cytology smears of BALF in the presence of a compatible clinical and chest imaging findings; (3) fungi were demonstrated by histopathological examination of lung biopsy specimens; (4) positive results of blood cultures in the presence of compatible clinical and chest imaging features. Legionella infection was considered when single serum indirect fluorescent antibody titer was ≧1:256.

### Oxygenation index

The arterial oxygen tension (PaO_2_) was analyzed with ABL III (Radiometer, Copenhagen, Denmark). Oxygenation index (PaO_2_/FiO_2_ ratio) was calculated as PaO_2_ divided by inspired oxygen fraction (FiO_2_).

### BALF and blood samples

The fiberoptic bronchoscope (Model BF20 or P20; Olympus, Tokyo, Japan) was wedged in the orifice of a lobar or segmental bronchus of the right middle lobe or lingular division or other appropriate location. Diagnostic BAL was done using three aliquots of a 50-mL sterile isotonic sodium chloride. Aspirates were pooled into a siliconized container and kept on ice during transport. Part of the retrieved BALF was subjected to Papanicolaou and Riu’s staining routinely. Some slides were stored for subsequent special staining if clinically indicated. A 10-mL sample of venous blood was drawn immediately after BAL. The supernatants of blood and BALF were frozen and stored at -70°C until analyzed.

### BALF cell analysis

BALF for cell analysis was filtered through two-layer sterile gauzes to remove mucus, and then cellular materials were sedimented by centrifugation (2500 rpm for 10 min at 4°C) and the supernatant was stored at -70°C for cytokine analysis later. The pellet was re-suspended in 1 ml phosphate buffered saline (PBS) for quantitative cell count. The cell differentials of BALF were measured by a hemocytometer (Hausser Scientific, Horsham, PA, USA) for counting at least 200 cells. For analysis of lymphocyte subpopulations 200 μl of cell suspensions (2×10^6^ cells) were incubated for 40 min at 4°C with 20 ml fluorescein isothiocyanate conjugated monoclonal antibody (Becton Dickinson, San Jose, Ca, USA). Following the incubation, 2 ml of cold PBS was added to the pellet and washed at 2500 rpm for 10 min at 4°C. Prepared BALF samples were examined by flow cytometry (FACS Calibur, Becton Dickinson, San Jose, CA, USA).

### Measurement of cytokines and biomarkers

The levels of cytokines in the supernatants of BALF and blood were measured by the commercially available enzyme-linked immunosorbent assay kits: interleukin (IL)-1β, tumor necrosis factor (TNF)-α, IL-8, monocyte chemoattractant protein (MCP)-1, IL-17, IL-10 and transforming growth factor (TGF)-β1, and IL-1 receptor antagonist (IL-1RA) (R&D Systems; Minneapolis, MN, USA). The levels of biomarkers in the supernatantsof BALF and blood were determined by commercial enzyme-linked immunosorbent assay kits according to the manufacturer’s instructions: high mobility group box 1 (HMGB1, HMGB1 ELISA kit II; Shino-Test Corporation, Tokyo); Krebs von den Lungen-6 (KL-6, Eisai, Tokyo, Japan); receptor for advanced glycation end product (RAGE, R&D Systems; Minneapolis, MN, USA); advanced glycation end product (AGE, Cell Biolabs Inc., San Diego, CA, USA); surfactant protein D (SPD, Biovendor, Modrice, Czech Republic). The lower limits of these variables were adapted as follows: IL-1β, 1 pg/ml; TNF-α, 4.4 pg/ml; IL-8, 3.5 pg/ml; IL-17, 15 pg/ml; MCP-1, 5.0 pg/ml; IL-10, 3.9 pg/ml; TGF-β1, 4.61 pg/ml; IL-1RA, 6.3 pg/ml; SPD, 0.2 ng/ml; HMGB-1, 1 ng/ml; RAGE, 4.12 pg/ml and KL-6, 201 U/ml.

### Estimating number of *Pneumocystis jirovecii*fungus in the lung

This technique was developed by Baughman and his coworkers [11]. Briefly, the BALF specimens 400 ul were spun onto glass slides, using a cytocentrifuge (Cytospin; Shandon Southern Instruments, Sewickley, PA, USA). The slides were air dried and then stained with Riu’s stain (modified May-Giemsa stain). The fungal load was semi-quantitatively measured by counting the number of clusters of *Pneumocystis jirovecii* in relation to 500 nucleated cells per slide.

### Measurement of β-D-glucan

The concentration of β-D-glucan was measured by Fungitell assay. It was performed according to the protocol supplied by the manufacturer (Associates of Cape Cod, MA, USA).

### Statistical analysis

Data were expressed as mean and SD or median and interquartile range (IQR), where appropriate. Non-parametric tests were used to analyze the variables because most of them were not normally distributed. Comparisons of the continuous variables between pure PJP and mixed PJP patients were made using the Mann–Whitney U test. Comparisons of the variables in pure PJP patients who were subdivided into two groups based on the variables including oxygenation index, the use of ventilator or survivor status were made using the Mann–Whitney U test. Multivariate analysis was performed using stepwise logistic regression analysis. Comparison for categorical variables between two groups was examined using Chi-squared test and/or Fisher exact, when appropriate. Significance was defined as p <0.05. Statistical analysis was performed using SPSS version 13 (SPSS, Chicago, IL, USA).

## Results

### Demographic characteristics, clinical features and cell profiles in BALF

The demographic characteristics and clinical data of 65 non-AIDS patients. With PJP divided into two groups are shown in Table 1. The oxygenation index, Acute Physiology and Chronic Health Evaluation II scores, intensive care unit (ICU) length of stay, the percentage of ICU admission, use of ventilator and death showed no significant differences between the pure PJP and mixed PJP patients. The concurrent pulmonary infections included bacteria in four cases, aspergillus in one case, CMV in nine cases, cryptococcus in two cases, CMV and aspergillus in one case, and CMV and legionella spp. in one case. The total cell count and cell differentials, and lymphocyte subpopulations in BALF are summarized in Table 2. There were no significant differences in total cell count, cell differentials and lymphocyte subpopulations between PJP patients with and without concurrent pulmonary infection.

**Table 1:** **Demographic and clinical characteristics of 65 patients with PJP**

	Pure PJP	Mixed PJP	P value
Gender(M/F)	25/22	9/9	0.818
Age, years	49.1 ± 16.0	50.6 ± 17.7	0.587
Underlying diseases			
Hematological malignancy s/p chemotherapy	6	2	
Allogeneic peripheral blood stem cell transplantation	3	2	
Solid organ transplantation (kidney/heart/liver/pancreas/pancreas-kidney)	19/4/2/1/1	3/0/0/0/0	
Connective tissue diseases with immunosuppresants	6	2	
Glomerulonephritis with immunosuppresants	3	6	
Prostate cancer with immunosuppresants	1	0	
Breast cancer with immunosuppresants	0	1	
Long-term steroid therapy	1	1	
DM	0	1	
PaO_2_/FiO_2_ before BAL	230.0(182.2;278.7)	245.4(158.3;322.9)	0.631
APACHE II scores	23.00(18.00;27.50)	23.50(20.25;26.50)	0.987
ICU admission, case number (%)	23(48.9)	12(66.7)	0.269
ICU length of stay (days)	19.0(7.3;24.0)	11.0(6.3;21.3)	0.456
Ventilator needed, case number (%)	24(51.1)	11(61.1)	0.667
Death, case number (%)	19(40.4)	10(55.6)	0.403

Data are expressed as case number and % in parenthesis or median IQR (25%;75%).

PJP = *Pneumocystis jirovecii* pneumonia; Mixed PJP = PJP with concurrent other pulmonary infections; PaO_2_ = arterial oxygen tension; FiO_2_ = fraction of inspired oxygen; ICU = intensive care unit; BAL = bronchoalveolar lavage; APACHE = Acute Physiology and Chronic Health Evaluation.

**Table 2:** **Comparisons of cell profile and lymphocyte subpopulations in BALF between pure and mixed PJP patients**

	Pure PJP (N =47)	Mixed PJP (N =18)	P value
Total cell count, 10^3^/ml	46.00(21.24;95.16)	30.00(18.27;115.07)	0.560
Macrophages, %	67.50(57.00;82.00)	64.25(42.63;71.38)	0.246
Macrophage count, 10^3^/ml	36.23(12.64;53.01)	18.88(10.19;60.15)	0.519
Lymphocytes, %	10.25(3.00;29.63)	7.50(1.88;20.38)	0.364
Lymphocyte count, 10^3^/ml	3.39(1.18;17.04)	2.03(1.01;3.47)	0.283
Neutrophils, %	10.50(4.88;20.88)	21.50(9.13;33.13)	0.06
Neutrophil count, 10^3^/ml	3.97(1.84;11.41)	7.84(1.46;25.07)	0.386
CD4/CD8 ratio	22.30(8.50;37.40)	11.50(4.80;22.63)	0.127
CD4+ cells, %	2.48(0.30;8.02)	0.42(0.06;5.73)	0.302
CD4+ cells, 10^3^/ml	31.80(15.80;59.70)	16.85(8.28;51.48)	0.384
CD8+ cells, %	4.67(0.55;13.19)	0.51(0.21;19.01)	0.356
CD8+ cells, 10^3^/ml	0.67(0.31;1.20)	0.31(0.18;1.95)	0.384

Data are expressed as median IQR (25%;75%).

BALF = Bronchoalveolar lavage fluid; PJP = *Pneumocystis jirovecii* pneumonia; Mixed PJP = PJP with concurrent other pulmonary infections.

The comparison was made by Mann–Whitney U test.

### Comparisons of BALF and blood cytokines between pure and mixed PJP patients

The blood IL-1β was not measured because of being undetectable or very low levels based on our previous experience. Comparisons of cytokines in BALF and blood samples between pure PJP and mixed PJP patients are given in Table 3. The levels of BALF anti-inflammatory cytokines including IL-10, TGF-β1 and IL-1RA showed no significant differences between the two groups.

**Table 3:** **Comparisons of cytokines in BALF and blood between pure and mixed PJP patients**

	Pure PJP (N =47)	Mixed PJP (N =18)	P value
**BALF**			
IL-1β, pg/ml	2.13(1.66;2.91)	4.59(1.92;24.45)	0.024
TNF-α, pg/ml	10.10(8.83;13.08)	12.24(10.09;23.84)	0.026
IL-8, pg/ml	46.30(31.80;175.30)	298.85(110.98;434.63)	0.001
IL-17, pg/ml	15.00(15.00;15.00)	15.00(15.00;15.00)	0.883
MCP-1, pg/ml	1048.50(250.81;2842.70)	1140.25(459.46;2171.44)	0.649
IL-10, pg/ml	3.90(3.90;7.70)	3.90(3.90;3.90)	0.152
TGF-β1, pg/ml	49.60(42.10;60.30)	46.55(44.88;59.33)	0.899
IL-1RA, pg/ml	1177.95(609.10;2816.00)	1682.15(1174.78;4496.43)	0.257
IL-1β/IL-10	0.48(0.35;0.56)	1.18(0.29;5.22)	0.018
TNF-α/IL-10	2.33(1.26;2.64)	2.80(2.43;3.87)	0.011
IL-8/IL-10	10.21(4.69;29.44)	53.60(24.49;85.10)	0.001
IL-17/IL-10	3.85(3.17;3.85)	3.85(3.85;3.85)	0.698
MCP-1/ IL-10	214.87(55.52;362.63)	176.11(117.81;375.97)	0.567
IL-1β/TGF-β1	0.04(0.02;0.05)	0.11(0.03;0.42)	0.002
TNF-α/TGF-β1	0.19(0.11;0.25)	0.25(0.22;0.39)	0.004
IL-8/TGF-β1	0.76(0.28;2.22)	4.85(2.47;9.99)	<0.001
IL-17/TGF-β1	0.31(0.26;0.37)	0.27(0.25;0.36)	0.570
MCP-1/TGF-β1	10.48(0.96;42.87)	18.54(10.85;39.91)	0.182
IL-1β/IL-1RA	0.00(0.00;0.00)	0.01(0.00.0.02)	0.048
TNF-α/IL-1RA	0.01(0.00;0.02)	0.01(0.00;0.02)	0.743
IL-8/IL-1RA	0.06(0.02;0.22)	0.11(0.05;0.76)	0.385
IL-17/IL-1RA	0.01(0.01;0.03)	0.01(0.00;0.02)	0.385
MCP-1/IL-1RA	0.99(0.23;1.52)	0.65(0.08;1.59)	0.905
**Blood**			
TNF-α, pg/ml	12.00(9.00;17.10)	12.25(7.55;20.68)	0.829
IL-8, pg/ml	30.41(18.78;53.40)	71.80(32.90;136.33)	0.008
IL-17, pg/ml	15.00(15.00; 15.00)	15.00(15.00; 15.00)	0.832
MCP-1, pg/ml	1271.70(402.90;2229.20)	1928.80(525.95;3496.50)	0.285
IL-10, pg/ml	19.90(10.40;37.50)	33.90(9.88;62.53)	0.226
TGF-β1, pg/ml	458.20(245.89;7768.70)	5996.50(259.08;7819.45)	0.288
IL-1RA, pg/ml	884.00(354.68;2348.75)	2160.40(402.80;6116.50)	0.229
IL-8/IL-10	1.73(0.86;2.79)	2.38(0.92;6.05)	0.131
IL-8/TGF-β1	0.06(0.00;0.21)	0.01(0.00;0.30)	0.895
TNF-α/IL-10	0.56(0.35;1.12)	0.35(0.19;1.19)	0.284
TNF-α/TGF-β1	0.02(0.00;0.07)	0.00(0.00;0.07)	0.215

Data are expressed as median IQR (25%;75%).

BALF = Bronchoalveolar lavage fluid; PJP = *Pneumocystis jirovecii* pneumonia; Mixed PJP = PJP with concurrent other pulmonary infections; IL = Interleukin; TGF-β1 = Transforming growth factor-β1; TNF-α = Tumor necrosis factor-α; MCP-1 = Monocyte chemoattractant protein-1; IL-1RA = IL-1 receptor antagonist.

The comparison was made by Mann–Whitney U test.

The values of BALF IL-1β, TNF-α, IL-8 and the ratios of IL-1β/IL-10, TNF-α/IL-10, IL-8/IL-10, IL-1β/TGF-β1, TNF-α/TGF-β1, IL-8/TGF-β1 and IL-1β/IL-1RA were significantly higher in mixed PJP than in pure PJP patients. The blood levels of IL-8 were significantly higher in mixed PJP than those in pure PJP patients.

### Comparisons of BALF and blood inflammatory biomarkers between pure and mixed PJP patients

No significant differences in the inflammatory biomarkers levels in BALF or blood were observed when pure PJP were compared with mixed PJP patients. The biomarkers included KL-6, SPD, HMGB-1, RAGE and AGE. The results are shown in Additional file 1: Table S1.

### Comparisons of cytokines and cytokine ratios in BALF and blood between concurrent bacterial and viral infection in mixed PJP patients

We compared pro-inflammatory cytokines, anti-inflammatory cytokines and ratio of pro-inflammatory cytokines/anti-inflammatory cytokines in both BALF and blood in mixed PJP patients who were divided into concurrent pulmonary bacterial and viral infection groups. The results showed no significant differences in the variables measured except for that BALF IL-1β/IL-1RA ratio were significantly higher in bacterial infection group. The results are shown in Additional file 2: Table S2.

### Comparisons of *Pneumocystis jirovecii*burden in the lung

The median number of *Pneumocystis jirovecii* in the lung was 3 (IQR, 1;7) in pure PJP and 2.5 (IQR, 1;12) in mixed PJP patients. There was no significant difference between the two groups. The median levels of BALF β-D-glucan in pure and mixed PJP patients were 203.30 (IQR, 148.30;442.40) and 260.60 (IQR, 134.40;623.40), respectively. The median levels of serum β-D-glucan in pure and mixed PJP patients were 213.00 (IQR, 19.90;784.50) and 215.65 (IQR, 75.45;7103.00), respectively. The values of BALF and serum β-D-glucan were not significantly different between the two groups.

### Comparisons of cytokines and pro-inflammatory/anti-inflammatory cytokine ratios between pure PJP patients divided into groups by oxygenation index (PaO_2_/FiO_2_), the use of ventilator and outcome

Comparisons between pure PJP patients with PaO_2_/FiO_2_ > 200 mmHg and PaO_2_/FiO_2_ ≤ 200 mmHg are shown in Table 4. Compared to the patients with PaO_2_/FiO_2_ > 200 mmHg, those with PaO_2_/FiO_2_ ≤ 200 mmHg had significantly higher BALF levels of IL-1RA, ratios of IL-1β/TGF-β1, TNF-α/TGF-β1, IL-8/TGF-β1 and MCP-1/TGF-β1, and had significantly higher blood levels of TGF-β1 and ratios of IL-8/IL-10 and TNF-α/IL-10. The ratios of IL-8/TGF-β1 and TNF-α/TGF-β1 in blood was significantly lower in those with PaO_2_/FiO_2_ ≤ 200 mmHg. However, after stepwise logistic regression analysis, none of these variables was independent factor.

**Table 4:** **Comparisons of pro-inflammatory and anti-inflammatory cytokines in BALF and blood between pure PJP patients with PaO**_**2**_ **/FiO**_**2**_ **≤ 200 and >200 mmHg**

	PaO _2_/FiO _2_>200 mmHg(N =24)	PaO _2_/FiO _2_ ≤ 200 mmHg(N =9)	P value*	P value ^#^
**BALF**				
IL-1β, pg/ml	2.24(1.85;2.86)	2.65(2.00;5.32)	0.414	
TNF-α, pg/ml	10.09(7.82;12.44)	13.08(9.52;17.82)	0.154	
IL-8, pg/ml	48.40(24.38;168.25)	175.30(35.00;342.90)	0.193	
IL-17, pg/ml	15.00(15.00;15.00)	15.00(15.00;15.00)	0.645	
MCP-1, pg/ml	1066.50(663.37;2593.31)	2453.16(715.00;4288.47)	0.254	
IL-10, pg/ml	4.25(3.90;8.93)	3.90(3.90;8.12)	0.414	
TGF-β1, pg/ml	53.40(45.45;128.30)	43.90(42.10;51.70)	0.150	
IL-1RA, pg/ml	709.70(463.30;1730.20)	1835.20(929.33;2816.00)	0.045	0.144
IL-1β/IL-10	0.41(0.29;0.57)	0.54(0.40;1.36)	0.238	
TNF-α/IL-10	2.33(1.19;2.63)	2.44(2.06;3.50)	0.179	
IL-8/IL-10	8.83(3.67;32.04)	28.22(8.78;53.10)	0.166	
IL-17/IL-10	3.85(2.84;3.85)	3.85(2.42;3.85)	0.547	
MCP-1/ IL-10	220.15(110.95;334.84)	629.02(170.28;914.57)	0.131	
IL-1β/TGF-β1	0.03(0.01;0.05)	0.06(0.03;0.13)	0.026	0.741
TNF-α/TGF-β1	0.16(0.03;0.22)	0.23(0.22;0.37)	0.003	0.421
IL-8/TGF-β1	0.63(0.16;1.84)	3.26(0.80;7.94)	0.032	0.787
IL-17/TGF-β1	0.29(0.27;0.33)	0.36(0.29;0.38)	0.121	
MCP-1/TGF-β1	14.13(4.40;34.32)	58.27(13.32;107.14)	0.032	0.158
IL-1β/IL-1RA	0.00(0.00;0.01)	0.00(0.00;0.00)	0.210	
TNF-α/IL-1RA	0.02(0.01;0.04)	0.01(0.00;0.01)	0.121	
IL-8/IL-1RA	0.18(0.02;0.99)	0.07(0.02;0.12)	0.374	
IL-17/IL-1RA	0.02(0.01;0.03)	0.01(0.01;0.02)	0.140	
MCP-1/IL-1RA	0.99(0.24;4.05)	1.48(0.46;1.52)	0.750	
**Blood**				
TNF-α, pg/ml	14.50(10.60;17.50)	12.70(8.50;24.20)	0.640	
IL-8, pg/ml	33.81(18.85;78.35)	41.20(21.60;90.55)	0.766	
IL-17, pg/ml	15.00(15.00;15.00)	15.00(15.00;15.00)	0.643	
MCP-1, pg/ml	1557.80(792.83;2655.05)	1382.60(318.75;2337.80)	0.512	
IL-10, pg/ml	33.40(12.25;56.93)	20.80(9.65;32.95)	0.111	
TGF-β1, pg/ml	255.15(168.10;3908.20)	3479.90(1020.85;10050.65)	0.014	0.892
IL-1RA, pg/ml	1087.90(297.10;2697.80)	701.80(286.90;2375.60)	0.938	
IL-8/IL-10	1.22(0.59;1.76)	2.79(1.14;4.14)	0.020	0.903
IL-8/TGF-β1	0.12(0.04;0.48)	0.02(0.01;0.04)	0.020	0.787
TNF-α/IL-10	0.35(0.17;0.93)	0.72(0.43;1.34)	0.035	0.872
TNF-α/TGF-β1	0.07(0.02;0.24)	0.00(0.00;0.03)	0.012	0.233

Data are expressed as median IQR (25%;75%).

PaO_2_/FiO_2_ = arterial oxygen tension/fraction of inspired oxygen; BALF = Bronchoalveolar lavage fluid; PJP = *Pneumocystis jirovecii* pneumonia; IL = Interleukin; TGF-β1 = Transforming growth factor-β1; TNF-α = Tumor necrosis factor-α; MCP-1 = Monocyte chemoattractant protein-1; IL-1RA = IL-1 receptor antagonist.

The comparison was made by Mann–Whitney U test.

P*: univariate analysis, P^#^: multivariate analysis.

Compared with pure PJP patients without ventilator, BALF levels of IL-8 were significantly higher and the values of IL-10 were markedly lower in pure PJP patients with ventilator. BALF ratios of IL-8/IL-10, IL-1β/TGF-β1, TNF-α/TGF-β1, IL-8/TGF-β1, MCP-1/TGF-β1 and MCP-1/IL-1RA, and blood levels of IL-8 and IL-10 and were significantly higher in pure PJP patients with ventilator. The TNF-α/IL-10 ratio in blood was significantly lower in those with ventilator (Table 5). However, after stepwise logistic regression analysis, only BALF IL-8 was the independent factor (p =0.023, 95% confident interval [CI] =0.000-0.004).

**Table 5:** **Comparisons of pro-inflammatory and anti-inflammatory cytokines in BALF and blood between pure PJP patients with and without ventilator**

	Without ventilator (N =22)	With ventilator (N =25)	P value*	P value ^#^
**BALF**				
IL-1β, pg/ml	2.23(1.73;2.91)	2.10(1.53;3.46)	0.915	
TNF-α, pg/ml	9.81(7.23;14.31)	10.30(9.09;12.84)	0.332	
IL-8, pg/ml	35.63(22.28;70.59)	59.90(32.95;249.65)	0.023	0.043
IL-17, pg/ml	15.00(15.00;15.00)	15.00(15.00;15.00)	0.876	
MCP-1, pg/ml	847.20(114.46;3158.55)	1048.50(326.90;2761.43)	0.565	
IL-10, pg/ml	5.33(3.90;9.25)	3.90(3.90;5.50)	0.049	0.954
TGF-β1, pg/ml	53.40(42.10;707.20)	48.80(42.10;55.10)	0.203	
IL-1RA, pg/ml	1323.10(566.70;3432.50)	1142.20(632.70;2303.10)	0.678	
IL-1β/IL-10	0.39(0.26;0.55)	0.49(0.37;0.57)	0.119	
TNF-α/IL-10	2.23(1.11;2.58)	2.51(2.06;2.71)	0.098	
IL-8/IL-10	7.24(3.31;12.42)	15.36(8.26;48.67)	0.004	0.733
IL-17/IL-10	3.85(3.12;3.85)	3.85(2.84;3.85)	0.981	
MCP-1/ IL-10	103.17(28.60;402.18)	262.10(74.72;397.89)	0.172	
IL-1β/TGF-β1	0.03(0.00;0.05)	0.04(0.03;0.07)	0.018	0.545
TNF-α/TGF-β1	0.13(0.01;0.25)	0.21(0.18;0.26)	0.017	0.321
IL-8/TGF-β1	0.47(0.07;1.02)	1.35(0.73;5.61)	<0.001	0.707
IL-17/TGF-β1	0.29(0.08;0.46)	0.31(0.27;0.36)	0.793	
MCP-1/TGF-β1	5.13(0.50;36.72)	21.51(5.32;56.99)	0.023	0.369
IL-1β/IL-1RA	0.00(0.00;0.01)	0.00(0.00;0.00)	0.649	
TNF-α/IL-1RA	0.01(0.00;0.02)	0.01(0.00;0.02)	0.756	
IL-8/IL-1RA	0.03(0.01;0.08)	0.10(0.02;0.40)	0.126	
IL-17/IL-1RA	0.02(0.00;0.03)	0.01(0.01;0.03)	0.943	
MCP-1/IL-1RA	0.24(0.03;0.70)	1.47(0.28;1.85)	0.011	0.582
**Blood**				
TNF-α, pg/ml	9.90(8.30;17.10)	12.45(10.20;17.15)	0.123	
IL-8, pg/ml	22.30(14.23;40.75)	42.70(22.95;105.15)	0.004	0.969
IL-17, pg/ml	15.00(15.00;15.00)	15.00(15.00;15.00)	0.866	
MCP-1, pg/ml	931.65(368.58;1719.33)	1382.60(459.80;2485.55)	0.354	
IL-10, pg/ml	11.21(9.33;21.60)	32.90(13.35;47.25)	0.006	0.938
TGF-β1, pg/ml	260.35(225.00;11506.09)	557.50(269.85;5782.80)	0.898	
IL-1RA, pg/ml	2114.30(693.50;3696.90)	701.80(286.90;2117.70)	0.169	
IL-8/IL-10	1.83(0.80;2.61)	1.71(0.94;2.81)	0.966	
IL-8/TGF-β1	0.04(0.00;0.15)	0.10(0.01;0.23)	0.159	
TNF-α/IL-10	1.10(0.65;1.31)	0.45(0.25;0.74)	0.022	0.160
TNF-α/TGF-β1	0.00(0.00;1.80)	0.03(0.00;0.07)	0.540	

Data are expressed as median IQR (25%;75%).

BALF = Bronchoalveolar lavage fluid; PJP = *Pneumocystis jirovecii* pneumonia; IL = Interleukin; TGF-β1 = Transforming growth factor-β1; TNF-α = Tumor necrosis factor-α; MCP-1 = Monocyte chemoattractant protein-1; IL-1RA = IL-1 receptor antagonist.

The comparison was made by Mann–Whitney U test.

P*: univariate analysis, P^#^: multivariate analysis.

Similar findings could be found in pure PJP patients who were divided into subgroups by clinical outcome. Compared with survivors, the non-survivors had significantly higher BALF values of IL-8 and ratios of IL-8/IL-10, IL-1β/TGF-β1, IL-8/TGF-β1, MCP-1/TGF-β1, IL-8/IL-1RA and MCP-1/IL-1RA, and significantly higher blood levels of IL-10 and IL-8. The blood level of IL-1RA was significantly lower in non-survivors (Table 6). However, after stepwise logistic regression analysis, only BALF IL-8/TGF-β1 was the independent factor (p =0.018, 95% CI =0.022-0.186).

**Table 6:** **Comparisons of pro-inflammatory and anti-inflammatory cytokines in BALF and blood between survivors and non-survivors of pure PJP patients**

	Survivor (N =28)	Non-survivor (N =19)	P value*	P value ^#^
**BALF**				
IL-1β, pg/ml	2.21(1.68;3.51)	2.00(1.50;2.91)	0.948	
TNF-α, pg/ml	10.07(7.98;14.05)	10.10(9.07;11.60)	0.761	
IL-8, pg/ml	35.63(22.60;79.26)	114.80(36.70;375.60)	0.010	0.694
IL-17, pg/ml	15.00(15.00;15.00)	15.00(15.00;15.00)	0.751	
MCP-1, pg/ml	736.49(218.78;2703.81)	1065.50(344.00;3069.70)	0.474	
IL-10, pg/ml	4.61(3.90;8.89)	3.90(3.90;4.40)	0.079	
TGF-β1, pg/ml	53.40(43.00;194.80)	48.80(41.20;54.13)	0.094	
IL-1RA, pg/ml	1589.90(669.20;3178.55)	1077.40(512.40;1870.50)	0.254	
IL-1β/IL-10	0.41(0.28;0.56)	0.49(0.36;0.57)	0.293	
TNF-α/IL-10	2.33(1.15;2.63)	2.51(2.20;2.71)	0.193	
IL-8/IL-10	8.26(3.66;13.75)	17.79(8.90;54.86)	0.002	0.735
IL-17/IL-10	3.85(3.48;3.85)	3.85(2.27;3.85)	0.451	
MCP-1/ IL-10	112.85(39.67;334.84)	273.21(79.44;433.15)	0.146	
IL-1β/TGF-β1	0.03(0.00;0.05)	0.04(0.03;0.06)	0.042	0.611
TNF-α/TGF-β1	0.15(0.01;0.26)	0.21(0.18;0.25)	0.091	
IL-8/TGF-β1	0.51(0.13;1.03)	1.56(0.71;7.73)	0.001	0.018
IL-17/TGF-β1	0.28(0.23;0.41)	0.31(0.27;0.36)	0.505	
MCP-1/TGF-β1	6.09(0.54;39.10)	21.57(5.49;58.27)	0.044	0.486
IL-1β/IL-1RA	0.00(0.00;0.00)	0.00(0.00;0.01)	0.451	
TNF-α/IL-1RA	0.01(0.00;0.02)	0.01(0.00;0.02)	0.354	
IL-8/IL-1RA	0.04(0.01;0.09)	0.12(0.03;0.53)	0.033	0.517
IL-17/IL-1RA	0.01(0.00;0.03)	0.01(0.01;0.03)	0.591	
MCP-1/IL-1RA	0.28(0.18;1.14)	1.48(0.46;4.43)	0.012	0.329
**Blood**				
TNF-α, pg/ml	11.10(8.56;18.20)	13.35(10.03;16.45)	0.335	
IL-8, pg/ml	23.65(16.53;41.55)	47.10(26.80;136.60)	0.002	0.397
IL-17, pg/ml	15.00(15.00;15.00)	15.00(15.00;15.00)	0.781	
MCP-1, pg/ml	1287.20(374.63;2124.20)	1014.60(529.10;2683.20)	0.537	
IL-10, pg/ml	14.05(8.78;26.62)	33.00(11.60;47.10)	0.018	0.952
TGF-β1, pg/ml	1020.85(245.89;10269.20)	423.40(205.00;6450.50)	0.515	
IL-1RA, pg/ml	1678.75(695.58;3536.63)	565.70(271.33;1594.93)	0.036	
IL-8/IL-10	1.74(0.81;2.45)	1.73(1.25;4.05)	0.423	
IL-8/TGF-β1	0.03(0.00;0.12)	0.12(0.02;0.24)	0.054	
TNF-α/IL-10	0.93(0.43;1.23)	0.45(0.24;0.80)	0.083	
TNF-α/TGF-β1	0.01(0.00;0.06)	0.05(0.00;0.09)	0.273	

Data are expressed as median IQR (25%;75%).

BALF = Bronchoalveolar lavage fluid; PJP = *Pneumocystis jirovecii* pneumonia; IL = Interleukin; TGF-β1 = Transforming growth factor-β1; TNF-α = Tumor necrosis factor-α; MCP-1 = Monocyte chemoattractant protein-1; IL-1RA = IL-1 receptor antagonist.

The comparison was made by Mann–Whitney U test.

P*:univariate analysis, P^#^:multivariate analysis.

### Comparisons of cytokines and pro-inflammatory/anti-inflammatory cytokine ratios between mixed PJP patients divided into groups by oxygenation index (PaO_2_/FiO_2_), the use of ventilator and outcome

There were no significant differences in BALF and blood levels of cytokines and pro-inflammatory/anti-inflammatory cytokine ratios between mixed PJP patients divided into two groups by oxygenation index and the use of ventilator. Compared with survived mixed PJP patients, BALF values of IL-1β, TGF-β1, IL-1β/IL-1RA ratio were significantly higher, and blood levels of TGF-β1 were markedly lower in the deceased ones.

## Discussion

Few studies discussed the difference in outcomes between pure PJP and PJP with concurrent pulmonary infection in non-AIDS immunocompromised patients. Most of the papers were focused on the CMV co-infection in HIV patients. However, the reported effect of concurrent CMV pneumonia on the outcome of AIDS patients with PJP was controversial. Some studies indicated that the morbidity and mortality showed no significant differences between HIV-infected PJP patients with or without concurrent CMV infection [12–15]. In contrast, AIDS patients with PJP who had co-infection with CMV pneumonia in other studies had higher mortality than those with PJP only [16, 17].

The role of concurrent CMV pneumonia in non-AIDS patients with PJP has not yet been well investigated. Miles et al. reported high mortality in non-HIV PJP patients irrespective of whether they had CMV pneumonia or not [15], but their case number was limited. Recently, Kim and coworkers reported that concomitant pulmonary CMV infection did not significantly affect the outcome of PJP with respect to the morbidity and mortality in non-HIV-infected patients with PJP [18]. To the best of our knowledge, this is the first study to compare the outcomes of non-AIDS PJP patients with or without concurrent pulmonary infections, including CMV pneumonia. Our results showed no significant differences in the morbidity and mortality between the two groups of patients (Table 1). Because the studied cases of PJP with concurrent pulmonary infection were limited and CMV pneumonia appeared to be the most common concurrent pulmonary infection, further studies with larger populations are needed to verify these issues.

Theoretically, exaggeration of pro-inflammatory or anti-inflammatory cytokine response may result in excessive inflammation leading to host tissue damage and affect the outcome of the patients. The results of our previous study indicated that as compared with normal lung group, PJP patients had significantly higher levels of pro-inflammatory cytokines and unvaried anti-inflammatory cytokines, and some pro-inflammatory/anti-inflammatory cytokine ratios were of value in assessing the severity of PJP and in predicting the outcome of the patients [10]. In the current study, the inflammatory cellular responses, as reflected by total cell counts, cell differentials and lymphocyte subpopulation in BALF, between pure PJP and mixed PJP patients showed no significant differences (Table 2). The levels of inflammatory biomarkers in BALF and blood also did not demonstrate significant differences between two groups of patients. An imbalance of pro-inflammatory/anti-inflammatory cytokines was shown to be more exacerbated in mixed PJP than in pure PJP patients (Table 3). However, our results failed to show significant differences in the morbidity and mortality between the two groups. It remains unclear why there should be limited impact of concurrent pulmonary infection on the cell profiles of BALF and related immune responses involved in PJP of non-AIDS immunocompromised patients. Further studies with larger populations are needed to verify these issues.

T helper (Th) 17 cells, a subset of T helper cells, appear to play an important role in fungal clearance and defense [19, 20]. Impaired IL-17 response was reported to be associated with decreased clearance of Candida and Aspergillus [21, 22]. However, the role of IL-17 response in pneumocystis infection has not yet been well investigated. The study conducted by Rudner et al. showed decreased levels of IL-17 in the lungs caused by IL-23 deficiency or by neutralizing anti-IL-17 antibody resulted in transient decrease (three weeks after infection) in clearance of pneumocystis organisms in mice [23]. One previous study suggested that interferon-γ was required to suppress Th17 differentiation, and Th17 response seemed to be ineffective to clear pneumocystis infection in immunocompromised mice [24]. The only IL-17 study in human subjects indicated that the levels of alveolar lining fluid IL-17 showed no significant difference between PJP infected patients and two PJP free control groups of patients with or without immunosuppression [25]. However, all the three groups studied included some patients with mixed pulmonary infections. The present study demonstrated that BALF levels of IL-17 in pure PJP and mixed PJP patients were at the lower limit except for one patient and showed no significant difference. Furthermore, the BALF level of IL-17 and IL-23 did not differ between normal controls and either pure PJP or mixed PJP patients (Additional file 3: Table S3). This is in contrast with the previous study showing the IL-23-IL-17 cytokine axis is involved in murine pneumocystis carinii pneumonia [23]. The possible reason is that some inhibitors might depress or neutralize the levels of IL-23 and IL-17. Another one is that IL-23 and IL-17 might occur earlier in the disease course and could not be detected later. The roles of IL-23/IL-17 in PJP and mixed PJP with other respiratory infection patients need further studies to explore.

It is of interest to see the effect of concurrent viral or bacterial infection on the immune dysregulation of PJP, and its clinical relevance in the non-AIDS immunocompromised patients with mixed PJP patients. The results showed no significant differences in the variables measured except for that BALF IL-1β/IL-1RA ratio were significantly higher in bacterial infection subgroup (Additional file 2: Table S2). Because the cases in each subgroup were limited in this study, further studies with larger populations are needed to verify the issues.

Our result showed values of BALF IL-8, IL-8/IL-10 ratio, IL-8/TGF-β1 ratio were significantly higher in pure PJP patients with the neede of mechanical ventilation and in deceased patients (Tables 5 and 6). After stepwise logistic regression analysis, BALF level of IL-8 was the independent factor for the need of mechanical ventilation and BALF level of IL-8/TGF-β1 ratio was responsible for the death in PJP of non-AIDS immunocompromised patients. Taken together, BALF inflammatory cytokines or inflammatory cytokines/anti-inflammatory cytokine ratios alone may be not strong enough to serve as prognostic factors. Some other factors including the entities of immunosuppression in non-AIDS immunocompromised patients, associated co-morbidity and severity of PJP at the diagnosis may be responsible for the need of mechanical ventilation or death of the patients. Further studies with larger populations are needed to verify these issues.

Despite failing to demonstrate the effect of concurrent pulmonary infection on the outcome of non-AIDS PJP patients, the present study confirmed that immune dysregulation did occur in PJP of non-AIDS immunocompromised patients [10]. Accordingly, it is highly suggested that steroid treatment may be indicated in the treatment of PJP of non-AIDS immunocompromised patients to damp the immune reconstruction syndrome or immune restoration disease, which is characterized by a condition of acute pulmonary compromise that is associated with return of immune function in immunocompromised patients with pulmonary infection who were clinically stable prior to the return of immune function, caused by pathogens of PJP [26, 27].

## Conclusions

In summary, concurrent infection may cause more severe immune dysregulation of PJP in non-AIDS immunocompromised patients, but has limited effect on the morbidity and mortality of PJP patients. Because of limited number of cases studied, further studies with larger populations are needed to verify these issues.

## Electronic supplementary material

Additional file 1: Table S1: Comparisons of inflammatory biomarkers in BALF and blood between pure and mixed PJP patients. (DOC 34 KB)

Additional file 2: Table S2: Comparisons of cytokines and cytokine ratios in BALF and blood between bacteria and virus subgroups in mixed PJP patients. (DOC 44 KB)

Additional file 3: Table S3: Comparisons of IL-17 and IL-23 in BALF among pure PJP patients, mixed PJP patients and normal lung controls. (DOC 32 KB)

## Abbreviations

PJP: *Pneumocystis jirovecii* pneumonia
AIDS: Acquired immunodeficiency syndrome
TNF: Tumor necrosis factor
IL: Interleukin
TGF: Transforming growth factor
IL-1RA: IL-1 receptor antagonist
BALF: Bronchoalveolar lavage fluid
CMV: Cytomegalovirus
PaO_2_: Arterial oxygen tension
FiO_2_: Inspired oxygen fraction
HMGB1: High mobility group box 1
KL-6: Krebs von den Lungen-6
RAGE: Receptor for advanced glycation end product
AGE: Advanced glycation end product
SPD: Surfactant protein D
ICU: Intensive care unit
Th: T helper.
