# Supplementary material for: The impact of concomitant pulmonary infection on immune dysregulation in Pneumocystis jirovecii pneumonia
Source: BMC Pulm Med. 2014 Nov 19;14:182. doi: 10.1186/1471-2466-14-182 (PMC4247696; doi:10.1186/1471-2466-14-182)
Supplement: Supplementary file 1 — Additional file 1: Table S1: Comparisons of inflammatory biomarkers in BALF and blood between pure and mixed PJP patients. (DOC 34 KB) [file 12890_2014_613_MOESM1_ESM.doc]

**Additional file 1**

**Supplemental table**

Table S1 Comparisons of inflammatory biomarkers in BALF and blood between pure and mixed PJP patients

|  | Pure PJP  (N = 47) | Mixed PJP  (N = 18) | P value |
| --- | --- | --- | --- |
| BALF  KL-6, U/ml  SPD, ng/ml  HMGB-1, ng/ml  RAGE, pg/ml  AGE, U/ml | 649.40(360.00; 1094.50)  573.30(315.40; 1234.40)  3.32(2.94; 4.10)  1450.41(482.75; 2473.10)  0.01(0.01; 0.01) | 1266.90(458.40;1873.83)  424.25(247.22; 681.02)  3.549(2.74; 4.24)  823.68(396.90; 1955.70)  0.01(0.01; 0.01) | 0.10  0.13  0.78  0.32  0.81 |
| Blood  KL-6, U/ml  SPD, ng/ml  HMGB-1, ng/ml  RAGE, pg/ml  AGE, U/ml | 1070.70(573.40; 2108.00)  284.50(169.25; 574.42)  3.09(2.30; 3.90)  874.03(449.70; 1435.07)  0.02(0.01; 0.02) | 1486.95(759.78;2624.05)  413.95(153.97; 639.10)  3.80(2.60; 5.50)  544.30(222.10; 883.50)  0.02(0.01; 0.02) | 0.19  0.60  0.07  0.09  0.86 |

Data are expressed as median IQR (25%;75%).

BALF = bronchoalveolar lavage fluid; PJP = *Pneumocystis jirovecii* pneumonia; Mixed PJP = PJP with concurrent other pulmonary infections; KL-6= Krebs von den Lungen-6; SPD=Surfactant protein D; HMGB-1=High mobility group box-1; RAGE= Receptor for advanced glycation end product; AGE= Advanced glycation end products.
