# Supplementary material for: The impact of concomitant pulmonary infection on immune dysregulation in Pneumocystis jirovecii pneumonia
Source: BMC Pulm Med. 2014 Nov 19;14:182. doi: 10.1186/1471-2466-14-182 (PMC4247696; doi:10.1186/1471-2466-14-182)
Supplement: Supplementary file 2 — Additional file 2: Table S2: Comparisons of cytokines and cytokine ratios in BALF and blood between bacteria and virus subgroups in mixed PJP patients. (DOC 44 KB) [file 12890_2014_613_MOESM2_ESM.doc]

**Additional file 2**

**Supplemental table**

Table S2 Comparisons of cytokines and cytokine ratios in BALF and blood between bacteria and virus subgroups in mixed PJP patients

|  | Bacteria  (N = 4) | Virus  (N = 9) | P value |
| --- | --- | --- | --- |
| BALF  IL-1β, pg/ml  TNF-α, pg/ml  IL-8, pg/ml  IL-17, pg/ml  MCP-1, pg/ml  IL-10, pg/ml  TGF-β1, pg/ml  IL-1RA, pg/ml  IL-1β/IL-10  TNF-α/IL-10  IL-8/IL-10  IL-17/IL-10  MCP-1/ IL-10  IL-1β/TGF-β1  TNF-α/TGF-β1  IL-8/TGF-β1  IL-17/TGF-β1  MCP-1/TGF-β1  IL-1β/IL-1RA  TNF-α/IL-1RA  IL-8/IL-1RA  IL-17/IL-1RA  MCP-1/IL-1RA | 25.90(7.93;78.00)  11.50(10.20;21.28)  309.05(191.25;1641.78)  15.00(15.00;15.00)  1365.25(676.24;2141.43)  3.90(3.90;3.90)  55.15(46.18;63.00)  1439.90(1429.60;4158.80)  6.64(2.03;20.00)  2.95(2.62;5.46)  79.24(49.04;420.97)  3.85(3.85; 3.85)  350.06(173.39;549.08)  0.49(0.14;1.28)  0.23(0.19;0.35)  5.77(3.75;26.49)  0.24(0.24;0.32)  26.15(13.23;36.23)  0.02(0.01;0.07)  0.01(0.00;0.02)  0.20(0.08;1.45)  0.01(0.00;0.01)  0.72(0.41;1.59) | 3.10(1.88;6.09)  11.90(10.08;15.03)  114.80(40.35;341.30)  15.00(15.00;15.00)  482.12(366.73;2065.80)  3.90(3.90;7.92)  45.80(40.25;58.10)  2233.10(810.20;5971.85)  0.79(0.24;1.56)  2.59(1.69;3.33)  28.74(9.04;57.83)  3.85 (3.07;66.92)  123.62(94.03;352.22)  0.06(0.03;0.16)  0.26(0.23;0.30)  2.92(1.01;7.40)  0.32(0.26;5.78)  11.75(6.91;41.71)  0.00(0.00;0.01)  0.01(0.00;0.03)  0.05(0.01;0.28)  0.01(0.00;0.28)  0.23(0.05;6.33) | 0.148  1.000  0.260  1.000 0.414  0.604 0.148  1.000  0.148  0.414  0.076  1.000  0.148  0.106  0.604  0.199  0.167  0.414  0.024  0.714  0.167  0.905  0.381 |
| Blood  TNF-α, pg/ml  IL-8, pg/ml  IL-17, pg/ml  MCP-1, pg/ml  IL-10, pg/ml  TGF-β1, pg/ml  IL-1RA, pg/ml  IL-8/IL-10  IL-8/TGF-β1  TNF-α/IL-10  TNF-α/TGF-β1 | 17.15(8.45;27.65)  68.85(31.30;689.08)  15.00(15.00;15.00)  1928.80(412.53;3718.75)  57.25(15.00;133.78)  474.40(225.48;5898.55)  6321.90(2160.40;10483.40)  2.60(0.72;9.64)  0.25(0.01;3.00)  0.38(0.19;0.75)  0.06(0.01;0.12) | 10.10(7.20;20.45)  77.20(40.60;148.85)  15.00(15.00;15.00)  1886.90(474.00;3563.40)  30.20(9.55;38.20)  6822.00(2762.25;12139.40)  2183.50(376.50;6116.50)  2.57(1.25;7.35)  0.01(0.00;0.14)  0.65(0.25;1.55)  0.00(0.00;0.04) | 0.710  0.825  1.000  1.000  0.260  0.106  0.500  0.503  0.260  0.503  0.330 |

Data are expressed as median IQR (25%;75%).

BALF = Bronchoalveolar lavage fluid; PJP = *Pneumocystis jirovecii* pneumonia; Mixed PcP = PJP with concurrent other pulmonary infections;

IL = Interleukin; TGF-β1 = Transforming growth factor-β1; TNF-α = Tumor necrosis factor-α; MCP-1 = Monocyte chemoattractant protein-1; IL-1RA= IL-1 receptor antagonist.

The comparison was made by Mann-Whitney U test.
