# Supplementary material for: The impact of concomitant pulmonary infection on immune dysregulation in Pneumocystis jirovecii pneumonia
Source: BMC Pulm Med. 2014 Nov 19;14:182. doi: 10.1186/1471-2466-14-182 (PMC4247696; doi:10.1186/1471-2466-14-182)
Supplement: Supplementary file 3 — Additional file 3: Table S3: Comparisons of IL-17 and IL-23 in BALF among pure PJP patients, mixed PJP patients and normal lung controls. (DOC 32 KB) [file 12890_2014_613_MOESM3_ESM.doc]

**Additional file 3**

**Supplemental table**

Table S3 Comparisons of IL-17 and IL-23 in BALF among pure PJP patients, mixed PJP patients and normal lung controls

|  | Pure PJP  (n=47) | Mixed PJP  (n=18) | Normal lung controls (n=11) | P1 | P2 | P3 |
| --- | --- | --- | --- | --- | --- | --- |
| IL-17 | 15.00(15.00;15.00) | 15.00(15.00;15.00) | 15.00(15.00;15.00) | 0.88 | 0.89 | 1.00 |
| IL-23 | 14.40(6.80;39.43) | 14.95(6.80;132.63) | 146.10(6.80;569.60) | 0.99 | 0.49 | 0.51 |

Data are expressed as median IQR (25%;75%). The two cytokines unit: pg/ml

BALF = Bronchoalveolar lavage fluid; PJP = *Pneumocystis jirovecii* pneumonia; Mixed PJP = PJP with concurrent other pulmonary infections; IL=Interleukin; P1= P value of comparison between pure PJP and mixed PJP patients; P2= P value of comparison between pure PJP patients and normal lung controls; P3= P value of comparison between mixed PJP patients and normal lung controls patients. Normal lung controls = Eleven subjects without abnormal findings on thoracic HRCT and pulmonary function testing. They were sarcoidosis in 9 and behcet’s disease with uveitis in 2.
